# Small studies may overestimate the effect sizes in critical care meta-analyses: a meta-epidemiological study

**DOI:** 10.1186/cc11919

**Published:** 2013-01-09

**Authors:** Zhongheng Zhang, Xiao Xu, Hongying Ni

**Affiliations:** 1 Department of Critical Care Medicine, Jinhua Municipal Central Hospital, 351 Mingyue Street, Jinhua City, Zhejiang 321004, PR China

## Abstract

**Introduction:**

Small-study effects refer to the fact that trials with limited sample sizes are more likely to report larger beneficial effects than large trials. However, this has never been investigated in critical care medicine. Thus, the present study aimed to examine the presence and extent of small-study effects in critical care medicine.

**Methods:**

Critical care meta-analyses involving randomized controlled trials and reported mortality as an outcome measure were considered eligible for the study. Component trials were classified as large (≥100 patients per arm) and small (<100 patients per arm) according to their sample sizes. Ratio of odds ratio (ROR) was calculated for each meta-analysis and then RORs were combined using a meta-analytic approach. ROR<1 indicated larger beneficial effect in small trials. Small and large trials were compared in methodological qualities including sequence generating, blinding, allocation concealment, intention to treat and sample size calculation.

**Results:**

A total of 27 critical care meta-analyses involving 317 trials were included. Of them, five meta-analyses showed statistically significant RORs <1, and other meta-analyses did not reach a statistical significance. Overall, the pooled ROR was 0.60 (95% CI: 0.53 to 0.68); the heterogeneity was moderate with an I^2 ^of 50.3% (chi-squared = 52.30; *P *= 0.002). Large trials showed significantly better reporting quality than small trials in terms of sequence generating, allocation concealment, blinding, intention to treat, sample size calculation and incomplete follow-up data.

**Conclusions:**

Small trials are more likely to report larger beneficial effects than large trials in critical care medicine, which could be partly explained by the lower methodological quality in small trials. Caution should be practiced in the interpretation of meta-analyses involving small trials.

## Introduction

Small-study effects refer to the pattern that small studies are more likely to report beneficial effect in the intervention arm, which was first described by Sterne *et al*. [1]. This effect can be explained, at least partly, by the combination of lower methodological quality of small studies and publication bias [2,3]. Typically, such small-study effects can be evaluated by funnel plot. Funnel plot depicts the effect size against the precision of the effect size. Small studies with effect sizes of wider standard deviations should widely and symmetrically distribute at the bottom of the plot, and large studies should cluster at top of the plot, making it the shape of an inverted funnel plot. If a funnel plot appears asymmetrical, publication bias is assumed to be present.

In critical care medicine, studies are conducted in intensive care units (ICU) where the number of beds is limited. Due to the nature of population and the care setting, the studies in critical care frequently have a small sample size. Meta-analysis is considered to be an important tool to combine the effect sizes of small trials, allowing more statistical power to detect the beneficial effects of a new intervention. However, according to meta-epidemiological studies conducted in other biomedical fields, interpretation of meta-analyses of small trials should be cautious, and such meta-analyses may overestimate the true effect of an intervention [3,4]. Small-study effect has been observed when examining meta-analysis with binary [3] and continuous outcomes [4]. In critical care medicine, small-study effects have never been quantitatively assessed. Thus, we conducted this systematic review of critical care meta-analyses in an attempt to examine the presence and extent of small-study effects in critical care medicine.

## Materials and methods

### Search strategy and study selection

Medline and Embase databases were searched from inception to August 2012. There was no language restriction. The core search terms consisted of critical care, mortality and meta-analysis (detailed search strategy is shown in Additional file 1). Inclusion criteria were as follows: critical care meta-analyses involving randomized controlled trial; the end points should include mortality; at least one component trial had more than 100 subjects per arm on average. Exclusion criteria were systematic reviews without meta-analysis; all component trials were exclusively large (sample sizes ≥100 per arm) or small trials (sample sizes <100 per arm); meta-analyses included duplicated component trials. If there were several meta-analyses addressing the same clinical issue, we included the most updated one. Two reviewers (XX and ZZ) independently assessed the literature and disagreement was settled by a third opinion (HN).

### Data extraction

The following data were extracted from eligible meta-analyses: the lead author of the study, year of publication, number of trials, treatment strategy in the experimental arm, proportion of large trials in each meta-analysis, effect size and corresponding 95% confidence interval (CI), heterogeneity as represented by I^2^. For each component trial, we extracted the following data: sequence generating, allocation concealment, blinding, incomplete follow-up data, intention-to-treat analysis, sample size calculation, and year of publication. Sequence generating was considered adequate when the trial reported the method to generate the randomization sequence (for example computer, randomization table). Allocation concealment was considered adequate when the investigator responsible for patient selection was unable to predict allocation of the next patient. The commonly used techniques included the use of central randomization or sequentially numbered, opaque and sealed envelopes. Blinding was considered adequate if the experimental and control interventions were described as indistinguishable by patients or investigators [5].

Small and large trials were distinguished by a cutoff of an average of 100 subjects per arm. For example, if a two-arm trial had 113 patients in one arm and 87 patients in the other, it was considered a large trial. This definition was somewhat arbitrary. However, a sample size of 200 patients allowed an 80% statistical power to detect an absolute difference of 20% for binary outcomes (assuming that the proportion in the control group was 50%) at two-sided α = 0.05. Another reason for this definition was that critical care trials were usually small, and a greater cutoff point would significantly reduce the number of meta-analyses that were eligible for the analysis.

### Statistical analysis

Treatment effects were expressed as odds ratio (OR) for mortality. The number of events and total number of patients in each arm were extracted for each component trial. An OR <1 indicated beneficial effect in the experimental arm. A standard logistic regression model was used to examine whether estimated treatment effects differ according to whether a trial is large or small [6,7]. Ratio of OR (ROR) was estimated from the regression model. ROR <1 indicates larger effect size in smaller studies and ROR >1 indicates larger effect size in large trials. ROR was calculated separately for each meta-analysis. These RORs were then combined using a meta-analytic approach. Inverse variance weighting and either fixed effect or random effects models were used to pool these RORs. Meta-analyses involving exclusively large or small trials were not included in our analysis and thus did not contribute to the analysis. Heterogeneity between trials was estimated using I^2^. A rough guide to the interpretation of I^2 ^can be as follows: 0 to 40% represents unimportant heterogeneity; 30% to 60% represents moderate heterogeneity; 50% to 90% represents substantial heterogeneity and 75% to 100% represents considerable heterogeneity [8]. To account for the difference of estimated effects between large and small trials, the qualities of study reporting including sequence generating, blinding, allocation concealment, incomplete follow-up data, sample size calculation and intention to treat were compared between large and small trials. The proportions of large and small trials were compared based on the year of publication before and after 2002. This was defined arbitrarily. However, we feel that multicenter large trials have increased rapidly in the last 10 years. We analyzed the association between sample size and treatment effects, stratified according to the significance of effect size and heterogeneity within each meta-analysis. All statistical analyses were performed using Stata software version 11.0 (StataCorp LP, College Station, TX, USA). Statistical significance was considered at two-sided *P *<0.05.

## Results

### Study selection and characteristics

**Figure 1:**
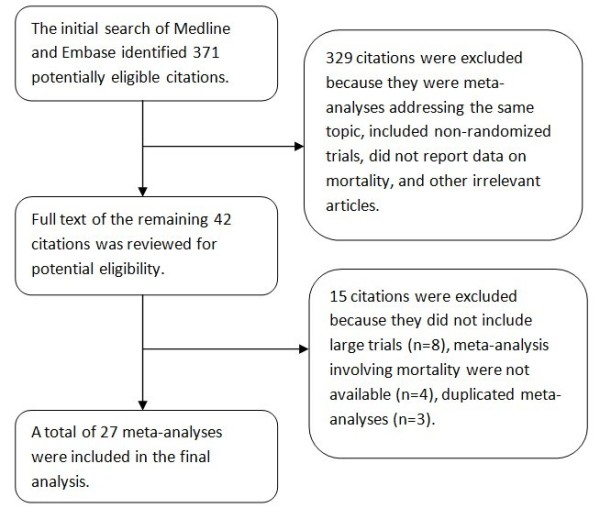
**Flow chart showing the selection of studies**.

Our initial search identified 371 citations. Of them, 329 were excluded by reviewing the title and abstract because they were duplicate meta-analyses, included non-randomized trials, did not report data on mortality, and other irrelevant articles. Full text of the remaining 42 citations was reviewed, of which 15 citations were excluded. In these excluded 15 citations, eight did not include large trials, study end point was not mortality in four meta-analyses, and three were duplicated meta-analyses. A total of 27 meta-analyses [9-35] involving randomized controlled trials were finally included in our analysis (Figure 1).

Characteristics of included meta-analyses are shown in Table 1. All meta-analyses were published after the year 2005. These meta-analyses covered all subspecialties of critical care medicine, including mechanical ventilation, sedation, fluid resuscitation, prevention of nosocomial infection, nutrition and critical care nephrology. For the number of component trials in each meta-analysis, we only counted those that reported mortality as an end point. Of note, because one meta-analysis [34] included seven trials conducted by Joachim Boldt that had been retracted [36], Boldt's trials were excluded from analysis. Eight meta-analyses [10,13,17,18,25,28,31,34] included only one large trial, and the meta-analyses by Zhongheng [35] included mostly large trials (83.3%). Most meta-analyses reported non-significant effect size, and only six meta-analyses [19,20,22,25,30,31] reported statistically significant effect sizes (for example mortality). Eight meta-analyses [12,15,16,20,21,30,32,35] reported high heterogeneity and the remaining 19 meta-analyses showed no significant heterogeneity among component trials. Seventeen meta-analyses [9-11,14,17,18,22-29,31,33,34] reported an I^2 ^of 0%, most of which were meta-analyses without significant effect sizes.

**Table 1:** Characteristics of included meta-analyses.

	Intervention	No. of studies ^II^	Event rate in control group (%)	Large trials (n, %)	Effect size of odds ratio (95% CI)	Heterogeneity (I^2^)
Abroug F 2011 [9]	Prone ventilation	7	37.3	3 (42.9)	0.91 (0.75, 1.1)	0%
Afshari A 2007 [10]	Antithrombin III	20	39.9	1 (5)	0.96 (0.89, 1.03)	0%
Afshari A 2011 [11]	Inhaled nitric oxide for ARDS	14	38.6	3 (21.4)	1.06 (0.93, 1.22)	0%
Annane D 2009 [12]	Corticosteroids	20	36.4	4 (20)	0.87 (0.74, 1.01)	44%
Augustes R 2011 [13]	Sedation interruption	5	37.2	1 (20)	0.84 (0.58, 1.21)	19.4%
Barkun AN 2012 [14]	PPI for stress-related mucosal bleeding	8	18.7	3 (37.5)	1.19 (0.84, 1.68)	0%
Blackwood B 2011 [15]	Weaning protocol	10	14.7	5 (50)	0.98 (0.48, 2.02)	57%
Burns KE 2011 [16]	Volume-limited ventilation	10	42.5	2 (20)	0.84 (0.7, 1.0)	43.1%
Delaney AP 2011 [17]	Albumin for resuscitation	17	33.8	1 (5.9)	0.82 (0.67, 1.0)	0%
Kopterides P 2010 [18]	Procalcitonin-guided	6	22.6	1 (16.7)	0.93 (0.69, 1.26)	0%
Landoni G 2010 [19]	Levosimendan	27	22.4	3 (11.1)	0.74 (0.62, 0.89)	11.3%
Laupland KB 2007 [20]	Polyclonal intravenous immunoglobulin	14	41.8	2 (14.3)	0.66 (0.53, 0.83)	53.8%
Marik PE 2008 [21]	Immunonutrition	24	25.8	5 (20.8)	0.85 (0.64, 1.13)	44%
Phoenix SI 2009 [22]	High PEEP	6	38.5	3 (50)	0.87 (0.78, 0.96)	0%
Pileggi C 2011 [23]	Selective decontamination of the digestive or respiratory tract	11	NR	6 (54.5)	1.1 (0.98, 1.24)	0%
Puskarich MA 2009 [24]	Glucose-insulin-potassium infusion	23	9	5 (21.7)	1.02 (0.93, 1.11)	0%
Serpa Neto A 2012 [25]	Vasopressin/terlipressin	9	50.3	1 (11.1)	0.87 (0.79, 0.96)	0%
Shah MR 2005 [26]	Pulmonary artery catheter	13	32.7	4 (30.8)	1.04 (0.9, 1.2)	0%
Shan L 2011 [27]	Intensive insulin therapy	5	34.7	2 (40)	0.98 (0.82, 1.16)	0%
Siempos II 2010 [28]	Probiotics	4	19.5	1 (25)	0.75 (0.47, 1.21)	0%
Tan JA 2010 [29]	Dexmedetomidine	16	8.7	3 (18.8)	0.85 (0.64, 1.17)	0%
Vasu TS 2012 [30]	Dopamine/norepinephrine	6	54.0	2 (33.3)	0.43 (0.26, 0.69)	65.4%
Visser J 2011 [31]	Micronutrient supplementation	10	28.8	1 (10)	0.78 (0.67, 0.90)	0%
Wang F 2011 [32]	Timing of tracheotomy	7	33.0	2 (28.6)	0.86 (0.65, 1.13)	45%
Wang F 2012 [33]	Subglottic secretion drainage	9	22.1	3 (33.3)	0.97 (0.84, 1.12)	0%
Zarychanski R 2009 [34]	Hydroxyethyl starch resuscitation	10	31.1	1 (10)	1.07 (0.85, 1.34)	0%
Zhongheng Z 2010 [35]	Dose of CRRT	6	45.4	5 (83.3)	0.91 (0.77, 1.08)	75%

^II^Only studies that reported mortality were included.

**Figure 2:**
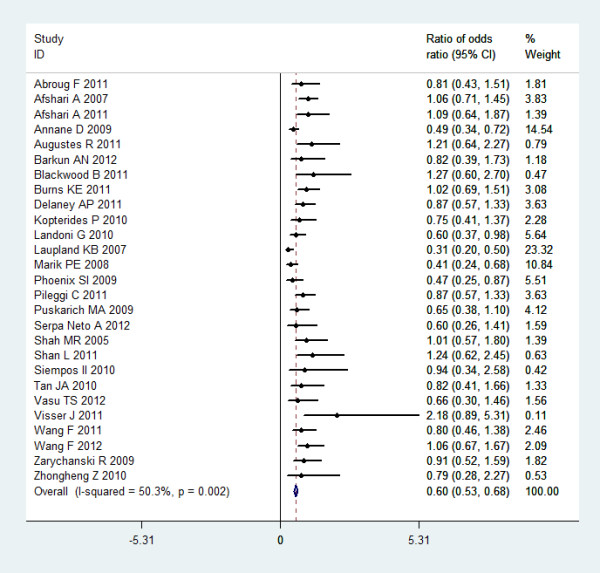
**Forest plot showing the ratio of odds ratio between small and large trials**.

RORs were estimated by logistic regression model separately for each meta-analysis (Figure 2). The RORs and relevant 95% CI are shown in the left column of Figure 2. Five meta-analyses [12,19-22] showed statistically significant RORs, indicating significantly larger beneficial effects in small studies; nine meta-analyses [10,11,13,15,16,26,27,31,33] showed RORs >1, but without statistical significance; the remaining 13 meta-analyses showed RORs <1, again without statistical significance. RORs were combined using a meta-analytic approach with inverse variance weighting. A fixed effect model was used to combine the RORs. Overall, the pooled ROR was 0.60 (95% CI: 0.53 to 0.68); the heterogeneity was moderate with an I^2 ^of 50.3% (chi-squared = 52.30; *P *= 0.002). Meta-regression was performed to test whether the observed RORs were dependent on the quality of meta-analyses. Covariates including concealment (*P *= 0.88), blinding (*P *= 0.82), intention to treat (*P *= 0.72), sequence generating (*P *= 0.48) and sample size calculation (*P *= 0.57) cannot explain the heterogeneity between meta-analyses.

To explore possible explanations for the difference of effect sizes between large and small trials, we compared reporting qualities of large and small trials (Table 2). As expected, the large trials showed significantly better reporting quality than small trials. More large trials were well conducted than small trials in terms of sequence generating, allocation concealment, blinding, intention to treat, sample size calculation and incomplete follow-up data. For instance, 82% of large trials explicitly reported allocation concealment, while only 51% of small trials reported this (*P *<0.001). Intention-to-treat analysis was used in 83% of the large trials, while only 52% of the small trials used this analysis (*P *<0.001). Sample size calculation was performed *a priori *in 88% of large trials and only 44% of small trials reported sample size calculation (*P *<0.001). Some 75% of large trials were published after the year 2002, while 52% of small trials were published after 2002 (*P *<0.001).

**Table 2:** Comparison of qualities between small and large trials in meta-analyses in critical care medicine.

	No. of patients in component trials		*P *value
	Large trials (>100 per arm, n = 73)	Small trials (<100 per arm, n = 244)	
Sequence generating			0.031
Adequate	29 (59%)	50 (41%)	
Inadequate/unclear	20 (41%)	72 (59%)	
Allocation concealment			<0.001
Adequate	56 (82%)	114 (51%)	
Inadequate/unclear	12 (18%)	111 (49%)	
Blinding			<0.001
Adequate	49 (67%)	99 (41%)	
Inadequate/unclear	24 (33%)	145 (59%)	
Incomplete follow-up data addressed			<0.001
Adequate	36 (86%)	56 (48%)	
Inadequate/unclear	6 (14%)	61 (52%)	
Intention to treat			<0.001
Yes	38 (83%)	79 (52%)	
No/unclear	8 (17%)	72 (48%)	
Sample size calculation			<0.001
Yes	29 (88%)	32 (44%)	
No/unclear	4 (12%)	40 (56%)	
Year of publication			<0.001
Before 2002	55 (75%)	127 (52%)	
After 2002	18 (25%)	117 (48%)	

We estimated ROR between large and small trials stratified according to the characteristics of individual meta-analysis (Table 3). Eight meta-analyses [16,17,19,20,22,25,30,31] reported significant effect sizes, and the pooled ROR was 0.49 (95%: 0.38 to 0.60). The remaining 19 meta-analyses reported insignificant effect sizes, and the pooled ROR was 0.69 (95%: 0.59 to 0.79). Stratified by heterogeneity, eight meta-analyses [12,15,16,20,21,30,32,35] reported high heterogeneity, their combined ROR was 0.46 (95%: 0.36 to 0.55); the remaining 19 meta-analyses showed a ROR of 0.79 (95% CI: 0.68 to 0.90). The result indicated that small-study effects were more prominent in meta-analyses of high heterogeneity.

**Table 3:** Ratio of odds ratio between large and small trials stratified by characteristics of meta-analyses.

Comparison	No. of meta-analyses	No. of trials	Ratio of odds ratio (95% CI)	Heterogeneity (I^2^)
Overall	27	317	0.60 (0.53-0.68)	50.3%
Significance of effect size				
Significant	8	99	0.49 (0.38-0.60)	62.9%
Non-significant	19	218	0.69 (0.59-0.79)	28.8%
Heterogeneity				
Low	19	220	0.79 (0.68-0.90)	0%
High	8	97	0.46 (0.36-0.55)	57.2%

## Discussion

This is the first meta-epidemiological study conducted in the field of critical care medicine to demonstrate smaller trials may overestimate treatment effect size. In this study, we included 27 meta-analyses of 317 randomized controlled trials covering all subspecialties of critical care medicine. The results showed that small trials were more likely to report larger estimated treatment effects compared with large trials, and this was more prominent in meta-analyses involving highly heterogeneous component trials. Furthermore, the small trials were of low quality in methodology compared with large trials, which may partly account for the small-study effects.

In a meta-epidemiological study in osteoarthritis [4], the authors employed the difference in effect sizes between large and small trials to explore the small-study effects. In line with the present study, they concluded that small trials were more likely to report larger beneficial treatment effects than large trials. However, in that study, the small trials were not statistically different from those of large trials in terms of blinding, intention-to-treat analysis, thus, the small-study effects cannot be fully explained by methodological quality. In osteoarthritis trials blinding is probably much more easily achieved because drugs can be made indistinguishable in appearance. Thus, small studies, usually with limited financial support, can also achieve good methodological quality. In contrast, in critical care medicine, blinding is sometimes impossible or complex due to the nature of intervention. Such interventions included pulmonary artery catheter, intensity of continuous renal replacement therapy, prone ventilation, and subglottic secretion drainage. In these situations, blinding may be difficult to achieve or only large trials with more planning and methodological support can make this possible. Thus, small trials in critical care medicine are of limited quality in design compared with large trials.

Possible explanations for the small-study effects have been explored. Kjaergard and colleagues [3] demonstrated that small studies with lower quality significantly exaggerated the intervention effect compared to large trials, while small trials with adequate sequence generating, allocation concealment and blinding did not differ from large trials. This is consistent with our findings that small studies had lower methodological quality compared with large trials. However, the impact of methodological quality such as allocation concealment and blinding varies according to different outcomes. A meta-epidemiological study involving 146 meta-analyses demonstrated that in trials with subjective outcomes the estimated effect sizes were exaggerated when there was inadequate concealment or blinding, while in trials with objective outcomes such as mortality, there was little evidence that inadequate concealment and lack of blinding would distort the estimated effect sizes [37]. This is in contrast with our findings, because we used mortality as an end point, but the result indicated that lack of blinding and inadequate allocation concealment might contribute to the exaggerated effect sizes in small trials. However, this is an unsettled question and there are other studies supporting our finding [38,39]. Most probably, individual quality measures such as blinding and allocation concealment are not consistently associated with the effect sizes across study areas, and each medical area should be specifically investigated [40]. Our analysis focused on the field of critical care medicine and showed, for the first time, a possible association of methodological quality with effect sizes.

There are several limitations in the present study. First, our study aims to investigate the small-study effect in critical care medicine. However, critical care is an extremely heterogeneous subspecialty that involves all varieties of diseases. Such heterogeneous nature may potentially impair the quality of the meta-epidemiological study. Second, the heterogeneity cannot be fully accounted for in the present analysis. We tried to explore the sources of heterogeneity by incorporating factors related to the quality of study design in meta-regression model, but failed to identify a significant covariate. We propose that the explainable factors may be those that cannot be readily accessible. Studies with negative results are less likely to be published than studies with positive results, particularly if such studies are small in sample size. As a result, small studies with negative result are less likely to be accepted into publication. If this is the case, it is not surprising that small studies are more likely to report beneficial effect. However, such kind of publication bias cannot be systematically investigated. Another explanation for the small-study effect may be that more rigorous implementation of interventions is performed in smaller studies.

## Conclusions

In conclusion, our study included 27 critical care meta-analyses involving all subspecialties in the field of critical care medicine. The result showed that small studies tended to report larger beneficial effects than large trials. Interpretation of meta-analyses of small trials should be cautious and sometimes definitive conclusions cannot be made until a large multicenter trial is conducted.

## Key messages

• Interpretation of critical care meta-analyses involving small studies should be cautious due to the small-study effects.

• Small-study effects may be attributable to the poor methodological quality of the small studies.

## Abbreviations

CI: confidence interval; ICU: intensive care unit; ROR: ratio of odds ratio.

## Competing interests

The authors declare that they have no competing interests.

## Authors' contributions

ZZ conceived the idea, collected data and drafted the manuscript; XX helped collect data and analyses; HN helped collect data, and analyze and interpret the results. All authors have read and approved the manuscript for publication.

## Supplementary Material

Additional file 1 **Search strategy**. The additional file shows the detailed search strategy performed in our study. Click here for file
