# Supplementary material for: Small studies may overestimate the effect sizes in critical care meta-analyses: a meta-epidemiological study
Source: Crit Care. 2013 Jan 9;17(1):R2. doi: 10.1186/cc11919 (PMC4056100; doi:10.1186/cc11919)
Supplement: Additional file 1 — Search strategy. The additional file shows the detailed search strategy performed in our study. [file cc11919-S1.DOCX]

**((((critical care[Title/Abstract]) OR intensive care medicine[Title/Abstract]) OR ICU[Title/Abstract]) OR intensive care[Title/Abstract]) OR critically ill[Title/Abstract]**

**mortality[Title/Abstract]**

**((meta-analysis[Title/Abstract]) OR systematic review[Title/Abstract]) OR metaanalysis[Title/Abstract]**

**(((randomized) OR controlled[Title/Abstract]) OR randomization[Title/Abstract]) OR randomly[Title/Abstract]**

**Initial search identified 371 citations**
